# Bacterial-Plant-Interactions: Approaches to Unravel the Biological Function of Bacterial Volatiles in the Rhizosphere

**DOI:** 10.3389/fmicb.2016.00108

**Published:** 2016-02-09

**Authors:** Marco Kai, Uta Effmert, Birgit Piechulla

**Affiliations:** Department of Biochemistry, Institute of Biological Science, University of RostockRostock, Germany

**Keywords:** bacterial volatiles, mVOC, plant growth promotion, plant growth inhibition, rhizobacteria

## Abstract

Rhizobacteria produce an enormous amount of volatile compounds, however, the function of these metabolites is scarcely understood. Investigations evaluating influences on plants performed in various laboratories using individually developed experimental setups revealed different and often contradictory results, e.g., ranging from a significant plant growth promotion to a dramatic suppression of plant development. In addition to these discrepancies, these test systems neglected properties and complexity of the rhizosphere. Therefore, to pursue further investigations of the role of bacterial volatiles in this underground habitat, the applied methods have to simulate its natural characteristics as much as possible. In this review, we will describe and discuss pros and cons of currently used bioassays, give insights into rhizosphere characteristics, and suggest improvements for test systems that would consider *in natura* conditions and would allow gaining further knowledge of the potential function and significance of rhizobacterial volatiles in plant life.

## Introduction

Volatile metabolites are important infochemicals mediating indispensable communication processes in all kingdoms of life (Hare, 2011; Dweck et al., 2015). The chemical nature of these small, lipophilic molecules enables plants attracting their pollinators or repelling pathogens, and animals finding mating partners (Hare, 2011; Mithöfer and Boland, 2012; Knollhoff and Heckel, 2014; Dweck et al., 2015). Current research indicates that volatiles released by bacteria play also a major role in multifarious microbial interactions, since these microbes release a wide range of various different volatiles, of which quite a few are capable to manipulate physiological processes in other bacteria, as well as in fungi and plants (Kai et al., 2009; Wenke et al., 2010, 2012; Effmert et al., 2012; Bitas et al., 2013; Audrain et al., 2015; Schmidt et al., 2015a).

Bacteria are omnipresent. They successfully occupy ecological niches as well as colonization hotspots like the plant rhizosphere. Plants release up to 40% of their photosynthetic fixed carbon through the roots into the surrounding area (Barber and Martin, 1976; Lynch and Whipps, 1991; Marschner, 1995; Hütsch et al., 2002). Due to this so called rhizodeposition, they attract a tremendous diversity of microorganisms (Perry et al., 2007). Bacteria preferably colonize the root itself (rhizoplane) and the adjunct soil zone (rhizosphere; Lenc et al., 2011; Bulgarelli et al., 2013; Reinhold-Hurek et al., 2015) representing thereby a crucial link between the plant roots and the surrounding soil. They take advantage of a constant flow of organic plant-based substrates, but in return promote plant growth by providing soluble inorganic nutrients and producing growth-promoting factors (Strzelczyk and Pokojska-Burdziej, 1984; Arshad and Frankenberger, 1988; O'Sullivan and O'Gara, 1992; van Rhijn and Vanderleyden, 1995; Spaink et al., 1998; Brimecombe et al., 2007; Nannipieri et al., 2007; Compant et al., 2010). The bacterial diversity and abundance and therefore the type of interactions with the plant root is shaped by the nature of rhizodeposits and soil properties. Rhizodepositions of course vary depending on species and growth stages of the plant, and environmental conditions (Bulgarelli et al., 2013). Soil properties are given e.g., by soil texture, chemical conditions, and moisture; many of them are strongly influenced by seasonal changes (Insam and Seewald, 2010; Bulgarelli et al., 2013). The interplay of all these factors causes a constant dynamic in the rhizo-ecosystem. The role of bacterial volatiles within this continuously changing community still remains mysterious.

Investigating these affairs in such a complex habitat is a challenge. Isolations of bacteria from the rhizosphere, their identifications (Berg et al., 2002), the determination of the volatiles produced (Kai et al., 2007, 2010; von Reuss et al., 2010), and particularly *in vitro* observations detecting the effects of rhizobacterial volatiles on plants (Ryu et al., 2003, 2004; Vespermann et al., 2007; Wenke et al., 2012) delivered first pieces of the puzzle. However, these data illustrate a dilemma. They have been mostly obtained from artificial and simplified bioassays and test systems, which do not or only partially reflect the complex conditions of the rhizosphere and therefore do not provide sustainable evidence whether microbial volatiles have a substantial impact on bacteria-plant-interactions in the rhizosphere *in natura*. In order to conceive the complexity of these interactions, additional experimental setups are needed to approach and simulate natural conditions and situations.

In this review, we will summarize currently used techniques and present the corresponding results. We will discuss the benefits, limitations, and pitfalls of these test systems and considering these facts, we would like to introduce ideas for improvements that might provide further insights into the volatile-based bacterial network within the rhizosphere and its implication for plant life.

## Different test systems—contrasting effects

The first documentation of bacterial volatile-mediated effects on plants was published by Cook and Stall (1969). It took more than 30 years until 2003 Ryu and coworkers seized the issue again and since then different test systems were used by different working groups. Table 1 gives an overview of all experimental systems that have been published so far. These systems can be characterized as follows: (i) setups that used passive diffusion or a directed airflow in order to transport the volatiles to the plant, (ii) target organs were the aerial parts of the plant or the roots, (iii) test system operating with an open or closed loop, and (iv) bacteria growing on different nutrients and matrices. Various combinations of these experimental setups were used. Up to now, in more than half of all existing studies the assays were operated with passive diffusion using divided Petri dishes (Table 1).

**Table 1:** **Methodical order of all experimental systems to study bacterial volatile-mediated effects on plants**.

**System**	**Effect**	**Bacteria**	**Plant**	**Medium**	**Compounds^a^**	**References**
**Petri dish bipartite** (parafilm sealed)	**Plant growth promotion** (total leaf surface area)	*Bacillus subtilis* GB03, *Bacillus amyloliquefaciens* IN937a, *Enterobacter cloacae* JM22	*Arabidopsis thaliana*	TSA	acetoin, 2,3-butanediol	Ryu et al., 2003
	**No visual effect**	*Bacillus pumilus* T4, *Bacillus pasteurii* C-9, *Serratia marcescens* 90-166, *Escherichia coli* DH5α			–	
	**Plant growth promotion** (induction of systemic resistance)	*Bacillus subtilis* GB03, *Bacillus amyloliquefaciens* IN937a, *Serratia marcescens* 90-166, *Bacillus pumilus* T4		TSA	2,3-butanediol (released from GB03 and IN937a)	Ryu et al., 2004
	**No induction of systemic resistance**	*Bacillus pasteurii* C9, *Enterobacter cloacae* JM22, *Pseudomonas fluorescen*s 89B61, *Bacillus pumilus* SE34			–	
	**Plant growth promotion** (induction of systemic resistance)	*Pseudomonas chlororaphis* O6		MS	2,3-butanediol	Han et al., 2006
	**Plant growth promotion** (regulation of auxin homeostasis and cell expansion)	*Bacillus subtilis* GB03		TSA	–	Zhang et al., 2007
	**Plant growth promotion** (shoot/root- fresh/dry- weight and length, leaf number)		*Ocimum basilicum*	MS	-	Banchio et al., 2009
	**Plant growth promotion** (dry weight)	*Serratia plymuthica* 4Rx13 (formely *odorifera*)	*Physcomitrella patens*	NB	CO_2_	Kai and Piechulla, 2010
	**Plant growth promotion** (shoot fresh weight)	*Bacillus megaterium* XTBG34	*Arabidopsis thaliana*	TSA	2-pentylfuran	Zou et al., 2010
	**Plant growth promotion** (shoot fresh weight)	*Bacillus cereus* B-569*, Burkholderia terricola* LMG 20594*, **Chromobacterium violaceum*** **CV0***, Cupriavidus necator, Escherichia coli, Stenotrophomonas rhizophila*	*Arabidopsis thaliana*	MR-VP	–	Blom et al., 2011a
		*Burkholderia lata*		LB		
		***Serratia plymuthica***		MS		
		*Burkholderia anthina* LMG 20980*, Burkholderia caledonica* LMG 19076*, Burkholderia caribensis* LMG 18531*, Burkholderia caryophylli, Burkholderia cepacia, Burkholderia thailandensis, Burkholderia sordidicola, Burkholderia hospita, Serratia proteamaculans*		MR-VP, LB		
		*Burkholderia gladioli, Burkholderia graminis, Burkholderia phenazinium, **Burkholderia phytofirmans**, Burkholderia sacchari, Cellulomonas uda, Serratia entomophilia, Serratia marcescens, Serratia plymuthica* HRO-C48		MR-VP, MS		
		*Burkholderia lata, Burkholderia glathei, **Pseudomonas aeruginosa**, **Pseudomonas chlororaphis**, **Pseudomonas putida***		MR-VP, Angle		
		*Burkholderia andropogonis, Burkholderia glumae, Burkholderia xenovorans, Pandoraea norimbergensis*		MR-VP, LB, MS	–	
		*Burkholderia kururiensis, Burkholderia tropica*		MR-VP, LB, Angle		
		*Burkholderia fungorum, **Burkholderia phenoliruptrix, Pseudomonas fluorescens*** **WCS 417r**		MR-VP, MS, Angle		
		*Burkholderia pyrrocinia*		MR-VP, LB, MS, Angle		
	**Plant growth inhibition** (shoot fresh weight)	***Burkholderia phenoliruptrix**, **Burkholderia phytofirmans**, **Chromobacterium violaceum*** **CV0***, **Pseudomonas aeruginosa**, **Pseudomonas chlororaphis**, **Pseudomonas fluorescens WCS 417r**, **Pseudomonas putida***		LB		
		*Limnobacter thiooxidans, **Serratia plymuthica*** **IC14**		MR-VP, LB		
	**Plant growth promotion** (biomass, lateral root number)	*Pseudomonas fluorescens* SS101	*Nicotiana tabacum*	King's medium B	13-tetradecadien-1-ol, 2-butanone, and 2-methyl-n-1-tridecene	Park et al., 2015
**Petri dish tripartite** (parafilm sealed)	**Plant growth inhibition** (shoot fresh weight)	*Pseudomonas fluorescens* CHA0, *Pseudomonas aeruginosa* (strains PA01a, PA01b, TBCF10839, PA14, TB, PUPa3), *Pseudomonas chlororaphis* subsp. *aureofaciens* ATCC 13985, *Serratia marcescens* MG1, *Serratia plymuthica* IC14	*Arabidopsis thaliana*	LB	HCN	Blom et al., 2011b
**Plastic container** (parafilm sealed)	**Plant growth promotion** (accumulation of starch in plant leaves)	*Bacillus subtilis* 168, *Escherichia coli* BW25113, *Pseudomonas syringae* (1448A9, 49a/90, PKs), *Salmonella enterica* LT2, *Agrobacterium tumefaciens* (EHA105, GV2260)	*Nicotiana tabacum, Solanum tuberosum, Zea mays, Horedum vulgare, Medicago sativa, Ocimum basilicum*	M9 minimal medium + 50 mM glucose	–	Ezquer et al., 2010
	**Plant growth promotion** (shoot/root- fresh/dry- weight and length, leaf number)	*Bacillus subtilis* GB03	*Ocimum basilicum*	MS		Banchio et al., 2009
	**Plant growth promotion** (shoot fresh weight, dry weight, rosette number, silique number, increased photo efficiency)	*Bacillus subtilis* GB03	*Arabidopsis thaliana*	TSA		Xie et al., 2009
**Petri dish bipartite** (parafilm non-sealed)	**Plant growth inhibition** (shoot fresh weight determination)	*Pseudomonas fluorescens* L13-6-12, *Pseudomonas trivialis* 3Re2-7, *Serratia plymuthica* 4Rx13 (formely *odorifera*), *Serratia plymuthica* 3Re4-18, *Serratia plymuthica* HRO-C48, *Stenotrophomonas maltophilia* R3089, *Stenotrophomonas rhizophila* P69	*Arabidopsis thaliana*	NB	–	Vespermann et al., 2007
	**Plant growth inhibition (**induction of H_2_O_2_ production)	*Serratia plymuthica* HRO-C48, *Stenotrophomonas maltophilia* R3089				Wenke et al., 2012
	**Plant growth promotion** (shoot fresh weight determination)	*Serratia plymuthica* 4Rx13 (formerly *odorifera*)		NBG	–	Weise et al., 2013
	**Plant growth inhibition** (shoot fresh weight determination)			NB	ammonia	
	**Plant growth inhibition** (dry weight determination)		*Physcomitrella patens*		-	Kai and Piechulla, 2010
Pots with **direct root inoculation**	**Plant growth promotion** (induction of systemic tolerance to drought)	*Pseudomonas chlororaphis* O6	*Arabidopsis thaliana*	peat: vermi-culite/perlite	2,3-butanediol	Cho et al., 2008
	**Plant growth promotion** (induction of systemic resistance)	*Bacillus subtilis* FB17		peat pellet	acetoin	Rudrappa et al., 2010
Petri dish placed underneath the soil at the bottom of a **plastic container**	**Plant growth promotion** (biomass, lateral root number determination)	*Pseudomonas fluorescens* SS101	*Nicotiana tabacum*	King's medium B	13-tetradecadien-1-ol, 2-butanone, and 2-methyl-n-1-tridecene	Park et al., 2015
**Dynamic air stream** targeting the aerial part	**Plant growth promotion** (shoot fresh weight determination)	*Serratia plymuthica* 4Rx13 (formerly *odorifera*)	*Arabidopsis thaliana*	NB	–	Kai et al., 2009
**Dynamic air stream** targeting the root						

a *Identified compounds that were shown to affect plant growth. Labeled in **bold** are bacterial strains that exhibited both positive and negative effects depending on the respective growth medium*.

### Passive diffusion systems

#### Closed systems

**Figure 1:**
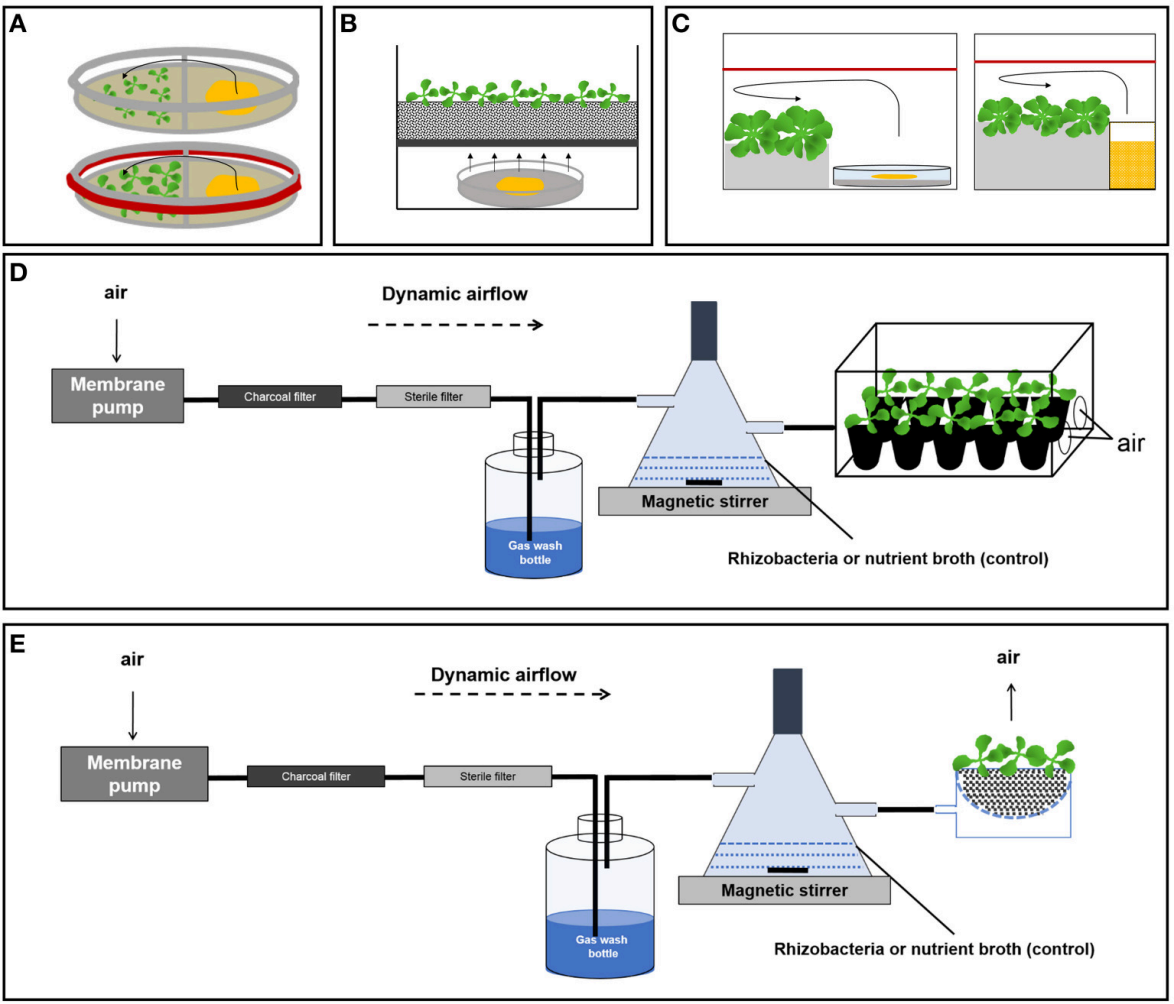
**Different bacterial volatile test systems. (A)** Passive diffusion in the Petri dish system (upper panel: open system, lower panel: closed system). **(B)** Passive diffusion targeting the roots. **(C)** Passive diffusion in container systems useful for older and bigger plants targeting the aerial plant parts (left: bacteria grown on Petri dishes, right: bacteria grown in liquid nutrient medium). **(D)** Dynamic air stream system targeting the aerial plant parts. **(E)** Dynamic air stream system targeting the roots.

The split Petri dish is a simple and the most favored system (Figure 1A). A barrier separates the dish into two or three compartments to ensure a physical separation of rhizobacteria and plants. The exchange of metabolites is facilitated solely via headspace. Primary target of volatiles is the aerial part of a plant. In order to prevent the escape of volatile metabolites, the Petri dishes were sealed with parafilm. This way, Ryu et al. (2003) could show that volatile compounds produced by plant growth promoting rhizobacteria dramatically increased the leaf surface area of *Arabidopsis thaliana* seedlings. The entire mixture of volatile metabolites as well as the fermentation products acetoin and 2R,3R-butanediol were shown to be responsible for this effect (Ryu et al., 2003). Both substances were found to trigger induced systemic resistance (Ryu et al., 2004). The data of Han et al. (2006) supported these results. An increase of the total leaf area of *A. thaliana* seedlings was also evident in experiments of Zhang et al. (2007), who additionally showed an influence of rhizobacterial volatiles on auxin homeostasis and cell expansion. Growth promotion was also observed in experiments with sealed split Petri dishes conducted by Banchio et al. (2009); Kai and Piechulla (2009); Ezquer et al. (2010); Kai and Piechulla (2010); Zou et al. (2010); Blom et al. (2011a,b); and Park et al. (2015). A variation of the sealed Petri dish setup is represented by systems that use a box-in-box strategy (Figure 1C). A little container like a glass vial or a Petri dish inoculated with bacteria was placed in a big sealed container hosting the plants. Xie et al. (2009); Banchio et al. (2009); and Ezquer et al. (2010) employed this method. In all experiments, plant growth promotion was observed.

#### Open systems

Omitting the parafilm changed the Petri dish setup toward an open system, which had a distinct impact on the test conditions (Figure 1A). Volatile compounds do not accumulate inside the compartments, which interestingly caused a dramatic growth inhibition of *A. thaliana* (Vespermann et al., 2007; Kai et al., 2008; Wenke et al., 2012; Weise et al., 2013). Kai and Piechulla (2010) directly compared the sealed and unsealed Petri dish system using the moss *Physcomitrella patens*. They showed that in co-cultivation with *Serratia plymuthica*, the moss gained biomass in sealed systems, while in open systems it suffered a dramatic loss.

In additional experiments (Supplementary Table 1, Velázquez-Becerra et al., 2011; Orozco-Mosqueda et al., 2013; Bailly et al., 2014; Zamioudis et al., 2015), it unfortunately remained unclear whether an open or closed system had been used. However, it should been mentioned that the plant growth was promoted in these studies. Only Bailly et al. (2014) showed growth inhibition using *Pseudomonas* strains, which was attributed to bacterial HCN production.

#### Systems targeting the roots

In order to simulate natural conditions, Park et al. (2015) placed a *Pseudomonas fluorescens* culture in a plastic container underneath a defined soil compartment containing growing *A. thaliana* seedlings (Figure 1B). Bacterial volatiles passively spread and diffused into the soil. After 3 weeks of cultivation, the authors observed significant growth stimulation with an increase of fresh weight of *A. thaliana*.

Another approach was introduced by Cho et al. (2008) and Rudrappa et al. (2010). Roots of *A. thaliana* seedlings were inoculated with a suspension of *Pseudomonas chlororaphis* and *Bacillus subtilis*, respectively. This direct contact of bacteria with the root system did not exclude the influence of non-volatile bacterial metabolites, however, it was shown that the volatile metabolite 2R,3R-butanediol produced by *P. chlororaphis* triggered the induction of systemic tolerance to drought, and acetoin emitted by *B. subtilis* induced systemic resistance.

### Dynamic air stream systems

A dynamic streaming system was used by Cook and Stall (1969). A consistent flow of purified air passed over several bacteria containing agar plates and subsequently reached the aerial part of plants such as *Capsicum* sp., *Nicotiana* sp., *Lycopersicum* sp., and *Brassica* sp. The volatile mixtures of *Xanthomonas vesicatoria, Xanthomonas campestris, Xanthomonas phaseoli, Erwinia carotovora, Erwinia amylovora, Pseudomonas cichorii, Pseudomonas tabaci*, and *P. fluorescens* induced necrosis in leaves of respective plants, whereby *X. vesicatoria* volatiles even killed *Capsicum annum*. Based on these results, it was assumed that volatiles might be associated with the hypersensitive response in plants (Cook and Stall, 1969).

Kai and Piechulla (2009) compared dynamic air stream systems that targeted (i) the aerial part as well as (ii) the roots of adult *A. thaliana* plants. They reported a considerable growth stimulation and increase in biomass. For system (i), they used a “mini” greenhouse for plant propagation (Figure 1D). Air enriched with volatiles of *S. plymuthica* grown in liquid culture was directed through the headspace of the growth container. For setting up system (ii), a glass bowl with a perforated base holding the plants was precisely positioned over a second glass bowl, which was designed to form a lower compartment equipped with an air inlet. Air enriched with volatile metabolites of *S. plymuthica* entered the lower compartment and escaped via the upper bowl through the soil thereby passing the roots of the plants (Figure 1E).

## *In vitro* test systems - pitfalls and limitations

### The experimental system matters

The overview of test systems (Table 1) illustrated a correlation between the setup and the outcome of the experiment. The most apparent difference was observed between closed and open compartment systems. This became most obvious considering the Petri dish systems. Studies using non-sealed plates revealed plant growth inhibition whereas almost all studies with sealed systems resulted in plant growth promotions. Solely, *Pseudomonas* strains were able to kill plants under both conditions. A split Petri dish setup is simple, inexpensive, easy to handle, allows a high throughput of samples, and assures a physical separation of producer and receiver. Besides these advantages, however, this test system suffers from some disadvantages. Sealing leads to an accumulation of metabolites even up to non-physiological/non-natural concentrations ultimately changing the micro-environment of the compartment and subsequently modifying the metabolism of the test organisms. Most prominent is the accumulation of CO_2_ due to the bacterial metabolism (Kai and Piechulla, 2009) and the elevated level of humidity due to transpiration of plants (Tholl and Röse, 2006). The CO_2_ content in the Petri dish can increase up to 10-fold compared to ambient concentrations; which most likely support plant growth in sealed Petri dishes (Kai and Piechulla, 2009). Non-sealed Petri dish systems avoid this accumulation, thus providing a completely different micro-environment.

Another aspect should be considered when the Petri dishes are used. The headspace is limited and therefore only young plant seedlings can be sampled. Their metabolism differs from that of adult plants (Jones et al., 2009). The model plant *A. thaliana* was often investigated, because of its small size, its short life cycle and the availability of myriads of mutants. For other test plants like *Nicotiana tabacum, Solanum tuberosum, Zea mays, Hordeum vulgare, Medicago sativa*, and *Ocimum basilicum* and for attempts to use adult plants, bigger growth containers were designed (box systems).

Common to both systems is the fact that the volatile metabolites are released into the headspace and subsequently primarily the aerial parts of the plants are exposed to bacterial volatiles (Figures 1A–D). The habitat of interest, however, is the part of the plant that grows underground. It may be argued that bacterial volatiles diffuse into the agar to target the roots, however, only rather hydrophilic volatiles permeate into the hydrophilic agar, while the lipophilic nature of most volatile metabolites obstructs a passage into the agar. Furthermore, compartmentalized systems, especially Petri dishes, do not meet different physiological demands of the test organisms. Despite the fact that the rhizosphere is a dark environment, bacteria as well as plant roots are exposed to light in the used experimental setups (Figures 1A–E).

Technical solutions that resolve some of these constraints allow a direct application of rhizobacterial volatiles to the roots (Cho et al., 2008; Kai and Piechulla, 2009; Rudrappa et al., 2010; Park et al., 2015). However, persistent in all systems is the fact that the production of rhizobacterial volatile metabolites strongly depends on the nutrient source. Already, Cook and Stall (1969) observed a nutrient-dependent effect, since only bacteria grown on nutrient agar (NA) or Kings B medium (KBM) caused necrosis in leaf tissues. Blom et al. (2011a) comprehensively investigated the influence of bacteria grown on different media. Subsequently they observed altered and contrasting effects on plant growth. The deleterious effect was caused by HCN produced by *Pseudomonas* strains grown on protein rich media (LB agar; Blom et al., 2011b). The principle of this finding was also supported by Weise et al. (2013). *S. plymuthica* produced NH_3_ only on protein enriched nutrient agar. This caused plant growth inhibition in open Petri dish systems whereas in sealed systems plant growth was promoted (Kai and Piechulla, 2009). Plants in unsealed systems were harmed by gaseous NH_3_ itself, due to plant medium alkalization and/or NH${}_{4}^{+}$ toxicity (Weise et al., 2013). In sealed dishes, the high CO_2_ concentration might promote formation of acidic HCO${}_{3}^{+}$ thereby preventing alkalization and consequently growth inhibition.

These examples show that compartment systems and especially split Petri dishes develop very fragile and vulnerable micro-environments for both bacteria and plants. This has to be considered when interpreting the results. In summary, different test conditions hamper a direct comparison of the results obtained. However, every setup represents a valuable test system that contributed to the overall picture of bacteria-plant-interactions.

### Soil matters

While the adherence of bacteria to the rhizoplane seems to be supported by biofilm formation (Rudrappa et al., 2008; Reinhold-Hurek et al., 2015), the growth of bacteria and the fate of volatiles within the rhizosphere depend considerably on soil conditions (Effmert et al., 2012; Burns et al., 2015). In most experimental setups, bacteria were grown on artificial medium such as solid agar or liquid media. Although these media might be vaguely reminiscent of biofilm substrates or water filled pores, so far the influence of physicochemical properties of soil has been neglected in most experimental approaches. This involves the influence of soil on the bacterial life as well as the production and distribution of bacterial volatiles. Solely direct inoculations of bacteria (Cho et al., 2008; Rudrappa et al., 2010) and purging of volatile enriched air into the soil (Kai and Piechulla, 2009; Park et al., 2015) to some extent considered effects of the natural underground habitat.

### Ecological relevance of rhizobacterial volatiles—quality and quantity matter

In many experimental setups, bacteria were inoculated into artificial media. These mostly nutrient rich conditions directed the metabolic activity of bacteria and thereby influenced the quality and quantity of the volatile metabolite synthesis. A nutrient enriched zone is in fact present on the root surface where root-derived organic compounds attract a diverse and specialized bacterial community. Nutritional conditions in more distant areas from roots might be different resulting in an altered microbial community (Garbeva et al., 2014; Reinhold-Hurek et al., 2015). Regarding this aspect, *in vitro* concentrations and the quality of the mixture of bacterial volatiles should be critically examined.

### Cell numbers matter (true controls matter)

An important aspect is whether the influence of bacteria on plant growth is a specific or a general phenomenon. Most studies present bacterial strains without any effect on plant growth as a corresponding negative control; e.g., the laboratory strain *E.coli* DH5α. However, due to different metabolic abilities to grow under the same nutritional conditions the growth rate and subsequently the concentration of volatile released differ between test and control strains. Images of split Petri dish setups of several studies illustrate these differences. Thus, volatiles produced by the control strain might be simply below the minimal affective concentration, e.g., different bacterial spot sizes implicating different bacterial growth rates (Ryu et al., 2003; Han et al., 2006; Kai et al., 2008; Zou et al., 2010; Blom et al., 2011a; Park et al., 2015). Bailly and Weisskopf (2012) already discussed this issue and assumed cell number dependent effects on plant growth. At a certain time point, slow and fast growing bacterial species appear in different growth phases thereby influencing the plant growth in different ways. Experiments performed by Blom et al. (2011a) confirmed this assumption. In addition, different cell densities might influence the regulation of the production of volatiles via quorum sensing (Kesarwani et al., 2011). As a consequence, the initial cell numbers of different bacterial species especially those of test and control strains should be adjusted as well as final cell numbers need to be determined and compared.

### Interactions matter

The physical separation of bacteria and plants represents an ambiguous aspect of setups that have been used so far. It allows for an exclusive investigation of effects of volatile-mediated interactions. Intra- and interspecific interactions, however, represent an intrinsic characteristic of the rhizosphere (Burns et al., 2015). The spectrum of bacterial volatiles will be influenced and even altered by bacterial and/or plant metabolites such as root exudates, infochemicals, or antibiotics, which do not have to be necessarily volatile. These metabolites might be continuously or only upon interaction produced and secreted. Interaction-induced allelochemical production and allelochemical-induced production of bacterial volatiles represent one of the most interesting aspects of rhizosphere investigations (Garbeva et al., 2011, 2014; Hol et al., 2015; Schulz-Bohm et al., 2015). The simulation of rhizodeposition might be realized by a defined nutrient composition of the bacterial medium (Blom et al., 2011a; Garbeva et al., 2014; Schulz-Bohm et al., 2015); the influence of allelochemicals on bacterial volatile production and the consequences for plant-bacterial interaction still awaits a substantial investigation.

## Characteristics of the habitat—key demands for novel experimental setups

*In vitro* test systems used so far are indispensable to get first insights into volatile-mediated effects of rhizobacteria on plants. Nevertheless, refined systems and novel approaches are necessary to dig deeper into the precise mechanisms of volatile-based bacteria-plant-interactions. Adequate experimental setups should really mimic or simulate as closely as possible rhizosphere conditions. These conditions shape evolving microbial communities and consequently the quality and quantity of volatiles. At the same time, the rhizosphere represents the matrix that determines the fate of volatile metabolites and facilitates or limits their diffusion. Matrix properties include the soil properties, chemical conditions (pH, aeration), and environmental factors (temperature, water content, darkness; Voroney and Heck, 2015). Traits that particularly influence the microbial communities are the nutrient status (root exudates, trace elements) and the interplay within the microbial community/population. The following section will briefly highlight these characteristics that have to be considered in order to develop suitable novel experimental setups.

### Soil properties, chemical parameters, and environmental factors

Soil is an aggregation of inorganic and organic particles, whereby the inorganic material is glued together with the organic matter (Voroney and Heck, 2015). The particle size of the inorganic particles varies between 0.002 and 2 mm resulting in different soil components including clay (below 0.002 mm), silt (0.05–0.002 mm), and sand (2–0.05 mm). The proportion of the three components determines the soil texture. Aggregates of the soil minerals of different size and organic materials cluster together forming the soil structure. About 35% (mineral soils) up to 90% (organic soils) of a soil volume can be taken up by pore space. Hereby, soil pores with a diameter below 10 μm (micropores) are important for the aqueous environment of bacteria, while soil pores below 5 μm in diameter are not colonized by microorganisms most likely due to impaired diffusion of nutrients. However, the gaseous diffusion and more specifically the diffusion of volatiles into micropores is supposed to be slow because of the water content. In contrast, macropores (diameter > 10 μm) facilitate rapid air and volatile diffusion. Effects of volatiles mediated over short distances should involve micropores, whereas effects over long distances probably require macropores. Furthermore, size and shape of pores determine their water and/or air content. Water is the universal factor in the rhizosphere influencing soil aeration, moisture, osmotic pressure, and pH or nature and amount of soluble substances available to or affecting organisms. While for soluble compounds water represents a perfect medium of transportation, it hampers diffusion of volatile metabolites due to their lipophilic nature. Aeration, which is driven by diffusion between the atmosphere and soil, again, promotes the emanation of volatiles. Diffusion through air filled pores is 10,000 times better than through water filled pores (Voroney and Heck, 2015). Closely connected to soil aeration is the availability of molecular oxygen (O_2_). O_2_ belongs to the most crucial factors for aerobic activity in soil. Due to the lower diffusion distance compared to the atmosphere, the partial pressure (pO_2_) in the topsoil is higher compared to the deeper regions (Glinski and Stepniewski, 1985; Stepniewski and Stepniewska, 2009) and considering soil aggregates, the pO_2_ diminishes from the outside to the center where even anoxic states are obtained (Sexstone et al., 1985; Zausig et al., 1993). The pO_2_ in the rhizosphere, depends on respiration processes and the diffusive O_2_ replenishment (Glinski and Stepniewski, 1985; Uteau et al., 2015). The respective oxygen status is the factor that clinches the switch from aerobic to anaerobic respiration in soil which fundamentally influences the metabolome and hence the volatile production. The fluctuation of temperature has to be considered as another fundamental factor of influence. Every organism has its own temperature optimum (Farrell and Rose, 1967). Bacteria, of course, can adapt to fluctuating temperatures, but this might be coupled with alterations of metabolism of the respective organisms, which may also lead to a different profile of volatiles. Furthermore, when temperatures reached threshold levels, for instance for mesophilic and thermophilic bacteria, the species diversity and abundance of communities shifted (Leven et al., 2007). Seasonally and/or diurnally changing temperatures should be therefore taken into account when natural conditions should be simulated (Voroney and Heck, 2015). Temperature, however, influences not only organisms. The decrease of temperature can cause an attenuated evaporation and diffusion of volatile molecules in the soil. The online screening of the soil/atmosphere exchange of volatiles conducted by Asensio et al. (2007) showed that the emission of some soil VOCs was enhanced due to increased soil temperatures.

### Nutritional conditions, bacterial growth, and developmental stages

Nutrient conditions within the rhizosphere are primarily regulated by the plants exuding excess photosynthetic products through the roots into the soil (Barber and Martin, 1976; Lynch and Whipps, 1991; Marschner, 1995; Hütsch et al., 2002). Thereby, the relative and absolute amounts of plant-derived nutrients in the rhizosphere vary with the plant species, plant age, and environmental conditions the plant has to cope with (e.g., soil properties, biotic, and abiotic stresses). Rhizodeposits include sugars, polysaccharides, amino acids, organic acids, fatty acids, and sterols (reviewed in Uren, 2007). Unfortunately, exudation of rhizodeposites was mostly studied *in vitro* on media other than soil, and therefore the *in vivo* exudation status of the rhizosphere and its change in time remains speculative (Uren, 2007). However, Bulgarelli et al. (2013) impressively showed that a variation of rhizodeposits caused an alteration of the plant root microbiome implicating a very dynamic production of bacterial volatiles. Besides this nutritional influence it can be assumed that due to bacterial growth and different developmental stages the bacterial metabolism and subsequently the formation of volatiles is changing. The production and effects of volatiles in dependence on bacterial developmental factors as well as growth stages, the formation of biofilms, generation of spores, or movement factors like swimming and swarming has so far not been in focus of the current research.

### Bacterial interactions in the rhizosphere

Bacterial interactions in the rhizosphere occur in three directions, (i) interaction of the bacteria with the plant (Bais et al., 2006; Rudrappa et al., 2008; Bednarek and Osbourn, 2009), (ii) the intra- and interspecific communication within the bacterial community (Ryan and Dow, 2008; Shank and Kolter, 2009; Garbeva et al., 2011; Tyk et al., 2014), and (iii) interaction with protozoa and metazoa (Matz and Kjelleberg, 2005; Ronn et al., 2012).

Plants release plenty of secondary metabolites, e.g., terpenes, flavonoids, glucosinolates, and phenylpropanoids into the rhizosphere (Dixon and Paiva, 1995; Rasmann et al., 2005; Van Dam et al., 2009; Bressan et al., 2009; Moore et al., 2014). Minor modifications in secondary metabolite level can have an important impact on soil microbial communities (Bressan et al., 2009). Flavonoids for instance are able to mimic quorum sensing (QS) molecules and thereby influencing the bacterial metabolism (Hassan and Mathesius, 2012). The production of 2-aminoacetophenone, a volatile metabolite produced by *Pseudomonas aeruginosa, Streptomyces* spp., and *Burkholderia ambifaria* is known to be QS regulated (Cox and Parker, 1979; Dickschat et al., 2005; Kesarwani et al., 2011; Groenhagen et al., 2013). Since it is predicted that many more volatiles are QS regulated, it is assumed that the interplay between plants and bacteria can change the pattern of volatile emission of bacteria. Thus, the QS system that controls basic processes of the bacterial life (e.g., biofilm formation and motility; Lowery et al., 2008) is likely to also affect the quality and quantity of volatiles. This is particularly important in highly competitive situations between different bacterial organisms that benefit from nutrient rich conditions in the rhizosphere. Moreover, bacteria evolved different strategies of antagonism including the release of antibiotics, lytic enzymes, siderophores, and toxins (Thomashow et al., 1990; O'Sullivan and O'Gara, 1992; Chernin et al., 1995; Pliego et al., 2008; and others). Driven by these manifold antagonistic properties and also due to the competition regarding water, nutrient and space, the bacteria react with the production of own weapons (Abrudan et al., 2015). For instance during interaction, *Streptomyces coelicolor* induced pigment production and hyphae formation in *B. subtilis* PY79 and simultaneously *B. subtilis* PY79 enhanced the production of cannibalism toxins in *S. coelicolor* (Watrous et al., 2012). In addition to antagonistic action also cooperation might affect emission of volatile metabolites. Metabolome profiling revealed that *B. megaterium* and *Ketogulonicigenium vulgare* cooperated by exchanging a number of metabolites (Zhou et al., 2011). This exchange increased the pool of own metabolites and hence different potential precursors of volatiles were available for the cooperating strains. It is therefore conceivable that the emission of volatiles might also alter due to bacteria-bacteria interaction. First data supporting this assumption were presented by Hol et al. (2015) and Schulz-Bohm et al. (2015). While Hol et al. (2015) showed that a non-random loss in bacteria communities reduced antifungal volatile production, Schulz-Bohm et al. (2015) could show that both microbial interactions and shifts in microbial community composition had a strong effect on the volatile emission. Likewise, bacterial interactions with fungi have to be considered. Splivallo et al. (2015) showed impressively that fruiting body associated bacteria contributed to the smell of the truffle *Tuber borchii* by synthesizing thiophene from a currently unknown precursors produced by *T. borchii*. Furthermore, Schmidt et al. (2015b) showed that *Collimonas pratensis* and *S. plymuthica* PRI-2C showed significant changes in their motility when exposed to fungal volatiles coming to the conclusion that bacteria are able to sense and respond to fungal volatiles (Schmidt et al., 2015b).

## Future test systems

Considering the complexity of the rhizosphere, it is eligible in a first step to use standardized conditions, such as sterilized sand inoculated with a single bacterial isolate or mixtures of bacterial species. Although plant growth on sand does not always reflect the most frequently used soil by plants, it has the advantage that variation in soil structure is limited compared to other soils. After these initial studies with sand, experimental setups should be approximated to more complex natural soil conditions. The second challenge is to match the diversity of the rhizobacterial community. Here the proposed strategy would be to start with one or a few bacterial species and gradually increase the number and variation of combinations to simulate the rhizosphere situation.

**Figure 2:**
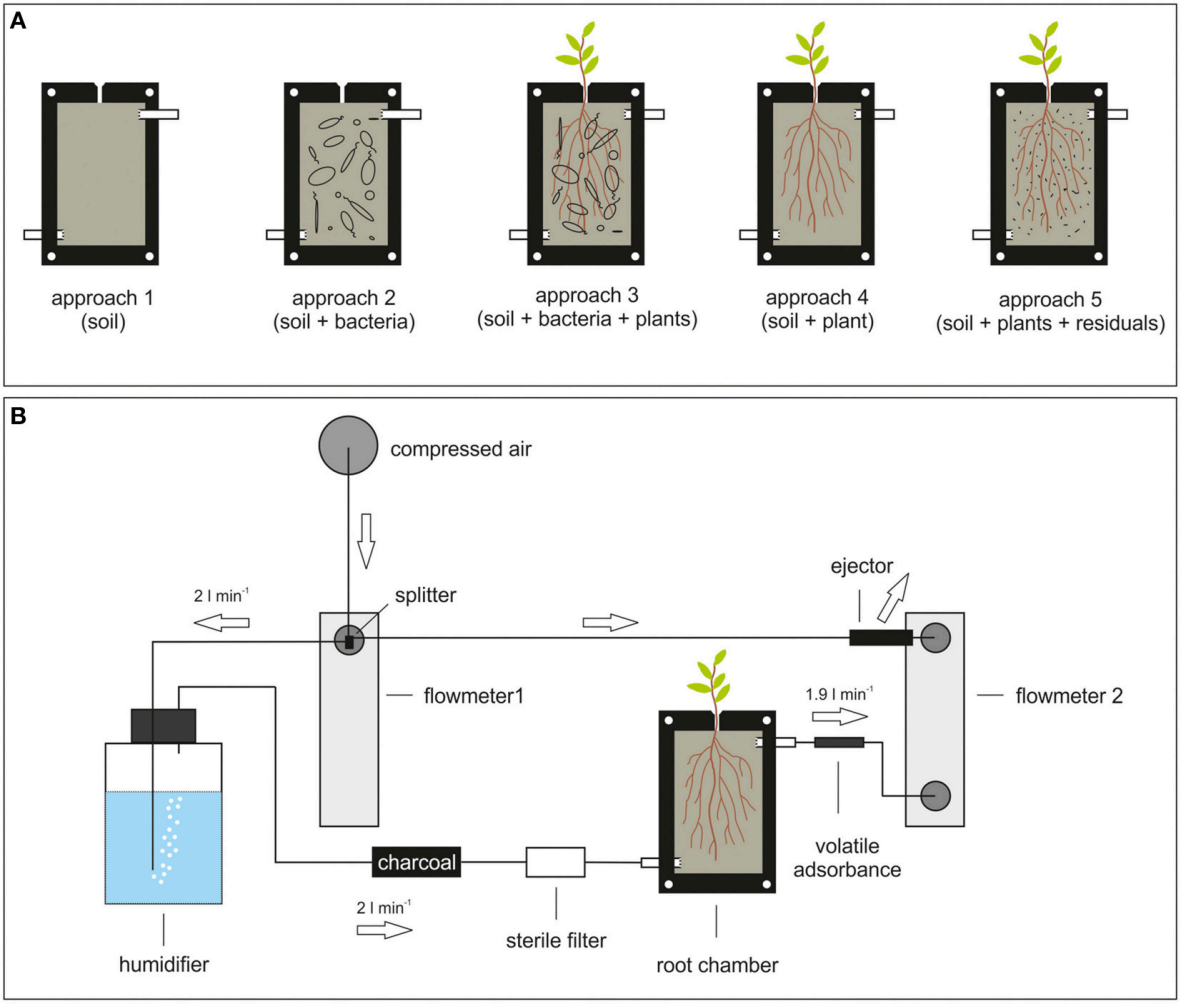
**Platform to analyze volatiles and volatile mediated effects. (A)** Root chamber. Several approaches are exemplified: 1. soil only; 2. soil and bacteria; 3. soil, bacteria, and plants; 4. soil and plants; 5. soil and bacterial residuals. **(B)** Design of a volatile-collection system using the root chamber. Arrows indicate direction of airflow. The flow of 2 l/min is exemplary indicated, since it always depends on the kind of adsorbent used. The splitter is separating the incoming airflow. On one site the air is humidified (gas washing bottle), and purified (charcoal and sterile filter) before passing into the root chamber. On the other site the ejector reverses the airflow that the volatile enriched air is pulled out of the root chamber over an adsorbent trap.

In order to investigate volatile compounds of root associated bacteria and to study their effects on plants, an experimental rhizosphere platform should be designed (Figure 2B). The core of the platform represents a root box equipped with an inlet and an outlet for air exchange (Figure 2A). The leakage of sand particles from the chamber is avoided by perforated barriers between the in- and outlet and the root box. The front plate of the box should be composed of transparent and inert Teflon® or glass material in order to observe root growth by e.g., eye or camera. On top of the box a small opening is the gateway for plant roots expanding into the box. The space inside the box can be filled with the matrix of choice regarding texture and composition. Before starting the experiment, the box as well as the matrix will be sterilized by gamma- or UV radiation, or by autoclaving. Surface sterilized seeds would be placed at the top puncture of the root box and upon germination the roots will push their way through the hole and develop their root network into the matrix.

The advantage of this setup is the possibility of arbitrary co-cultivation and co-development of (various) bacterial species with the plant root. The correct assignment of emitted volatiles either to the plant root or bacterial species has to be performed by differential analysis of the several setups (soil only, bacteria only, plant only, bacteria, and plant and so on; Figure 2A). Beside the characterization of plant growth parameter (shoot- and root fresh weight, plant omics) under various different conditions, a continuous measurement of volatile emission of appropriate time intervals can be established. Thereby, it should be distinguished between volatiles that bind to soil particles or aggregates by using *in situ* polydimethylsiloxane micro-extraction (Eilers et al., 2015) and volatiles that do not bind to soil by passing air through the root box and further over an adsorbent trap (Figure 2B). The above mentioned differential analysis would provide information about plant growth in combination with data about the status of volatiles in the root system. A similar approach was introduced by Eilers et al. (2015) originally developed for Dandelion (*Taraxacum* sect. *ruderalia*) root volatiles. This system aimed to simply and inexpensively detect rhizosphere chemicals at experimentally less disturbed conditions. Therefore, it could be adapted in order to use it for bacteria-plant interaction. Nevertheless, since the assignment of volatiles to the producer in these systems is still difficult, there is a need to verify specificity of volatile-mediated effects by evaluating the obtained data in the compartmented test systems *in vitro*. These verifications must include the check for profiles of bacterial volatiles as well as the application of single compounds/mixtures of volatiles. Such a combinatory approach of the different test systems and techniques will help to understand the volatile-mediated effects on plant growth.

## Conclusions

Recent advances have enhanced our understanding that small volatile molecules emitted by bacteria can have dramatic effects on the growth and development of plants. These observations were mainly based on various different experimental setups, often revealing discrepancies between results. In addition, most test systems neglected properties and complexity of the rhizosphere. In order to go beyond the search for potential effects and to evaluate the significance of rhizobacterial volatiles *in situ*, setups that mimic the rhizosphere, that allow for a combination of various bacterial species and if desired other microorganisms, that assure for the usage of different soil matrices, and that enable *in situ*-volatile collections, the root box embedded into the so called rhizosphere platform could be considered to be a first step into this direction.

## Author contributions

MK designed research; MK, UE, and BP wrote the paper.

### Conflict of interest statement

The authors declare that the research was conducted in the absence of any commercial or financial relationships that could be construed as a potential conflict of interest.

## Supplementary material

Supplementary Table 1 **Investigations in which remained unclear whether an open or closed system had been used**. ^1^Identified compounds that were shown to affect plant growth.
