# Supplementary material for: Bacterial-Plant-Interactions: Approaches to Unravel the Biological Function of Bacterial Volatiles in the Rhizosphere
Source: Front Microbiol. 2016 Feb 9;7:108. doi: 10.3389/fmicb.2016.00108 (PMC4746483; doi:10.3389/fmicb.2016.00108)
Supplement: Supplementary Table 1 — Investigations in which remained unclear whether an open or closed system had been used. 1Identified compounds that were shown to affect plant growth. [file Table1.docx]

| **System** | **Effect** | **Bacteria** | **Plant** | **Medium** | **Compounds^1^** | **Reference** |
| --- | --- | --- | --- | --- | --- | --- |
| **Petri dish bipartite** | **plant growth promotion** (plant freshweight, increase of lateral roots, decrease of taproot growth | *Arthrobacter agilis* UMCV2 | *Medicago sativa* | LB, NA | dimethylhexa-decylamine (8µM) | Velázquez-Becerra et al., 2011 |
|  | **plant growth promotion** (induction of systemic resistance and plant survival under conditions of limited iron availability) | *Pseudomonas simiae* WCS417 | *Arabidopsis thaliana* | King's medium B | - | Zamioudis et al., 2015 |
| **vertical plates** with bacteria placed in the middle of a stainless steel ring | **plant growth promotion** (biomass, lateral root number determination) | *Escherichia coli, Burkholderia anthina, Burkholderia caledonica, Burkholderia caribensis, Pseudomonas putida* ISOf (only biomass promotion) | *Arabidopsis thaliana* | LB | indole | Bailly et al., 2014 |
|  | **plant growth inhibition** (biomass,lateral root number determination) | *Pseudomonas chlororaphis, Pseudomonas aeruginosa PUPa3, Pseudomonas fluorescens* WCS417r |  |  | HCN |  |
| **glass flask** with bacteria placed in a glass vial | **plant growth promotion** (induction of iron acquisition) | *Arthrobacter agilis* UMCV2 | *Medicago sativa* | NA | dimethylhexa-decylamine (8µM) | Orozco-Mosqueda et al., 2013 |
